# Game Insight Skills as a Predictor of Talent for Youth Soccer Players

**DOI:** 10.3389/fspor.2020.609112

**Published:** 2021-01-15

**Authors:** Tom de Joode, Drewes J. J. Tebbes, Geert J. P. Savelsbergh

**Affiliations:** ^1^Department of Human Movement Sciences, Vrije Universiteit Amsterdam, Amsterdam Movement Sciences, Amsterdam, Netherlands; ^2^Institute of Brain and Behavior, Amsterdam, Netherlands; ^3^Faculty of Sports and Nutrition, Amsterdam University of Applied Sciences, Amsterdam, Netherlands

**Keywords:** perceptual-cognitive skills, game insight indicator, talent identification, youth elite soccer players, occlusion task, anticipation, decision making

## Abstract

Perceptual–cognitive skills are found to be important factors for soccer players. The aim of this study was, therefore, to find within-group differences for game insight in an elite group of youth soccer players by means of a Game Insight inDicator (GID). In addition, the prospective value of perceptual–cognitive skills was examined by following the trajectory of the participants. The GID consisted of film clips that show game situations. The task of the players was to predict the trajectory and destination of the ball and move toward the correct position to receive the pass of a teammate. The film clips stopped 80 ms before, at, and 80 ms after the football contact of a teammate. We also sought to validate the GID against game performance. Participants were talented soccer players 11–13 years old and playing at the elite level for their age. Based on eight independent elite-coach judgments, two groups were created: highly talented players (HT) and less talented players (LT). The coach ratings were supported by a significant difference between the two groups based on the objective notational analysis of their game performance in 4 vs. 4 and 11 vs. 11 matches. With respect to the GID, a significant interaction effect for the groups (HT vs. LT) by occlusion time (−80, 0, and +80 ms) was found, showing that the HT performs better than the LT in 0 and +80 ms condition. In addition, GID scores were compared with soccer levels at the mean age of 19 years. Longitudinal data did not show significant differences between elite and sub-elite. Overall, the GID was found to be a valid and useful indicator for players anticipating the ball's trajectory and destination at age 11–13 years but failed to predict the players' level at age 19 years. The latter indicates how difficult it is to predict talent development.

## Introduction

Most ball sports require the ability to intercept or pass the ball. To succeed, one has to be capable of anticipating the ball's trajectory and destination. When visual information available for players is limited, they have to rely even more on their capability to extract relevant cues from the limited amount of visual information available. This skilled perception is found to be a valid differentiator between expert and less-expert players. Major findings from several studies have shown that expert athletes can extract more relevant information from pre-ball flight cues than their less-expert counterparts (Mann et al., 2007; Savelsbergh et al., 2010). Thereby occlusion of sport-specific video, occluded at or near-final ball contact, can differentiate between expert and less-expert soccer players when the players are asked to follow the direction of the ball in the video (Savelsbergh et al., 2002). The research paradigm using the temporal occlusion of visual information was first introduced by Abernethy and Russell (1987) in squash to investigate the level of anticipation between expert and non-expert players. Later studies have reported that the discrepancy of anticipation capacity between expert and non-expert players also holds in many other ball sports such as tennis, baseball, cricket, and soccer (Houlston and Lowes, 1993; French et al., 1995; McPherson, 2000; Savelsbergh et al., 2002).

Perceptual–cognitive skills are reported to be crucial factors for soccer players (Roca et al., 2012). Furthermore, the literature on the occlusion paradigm suggests that perceptual–cognitive skills might be highly relevant for talent identification (TID). TID aims to recognize players in sports who will be successful in the future (Williams and Reilly, 2000). Literature has shown that this “talent” is something that does not remain stable and evolves with experience or expertise (Abbott and Collins, 2004; Vaeyens et al., 2008). Consequentially, the use of physiological measures such as height or sprint speed for TID might be affected by improper judgment. These improper judgments are developed by pitfalls in TID, such as the relative age effect and selection biases (Vaeyens et al., 2008; Christensen, 2009). Shifting toward psychological predictors of talent relatively less affected by latter pitfalls is more promising (Mann et al., 2017; Murr et al., 2018). For instance, as players increase with age, the general standard of skills and technical ability keeps getting better. This would mean that the technical skills of the successful players who reach the top would be extremely high, and the distinguishing factor of experts and novices on that level might not be in technical expertise but expertise in the mental aspect of the game (Woods et al., 2016a).

Perceptual–cognitive skills are typically measured with verbal or notational measures (Abernethy and Russell, 1987; Savelsbergh et al., 2002; Kannekens et al., 2009; Woods et al., 2016b). Earlier research found, for instance, that soccer players who scored high on the Tactical Skills Inventory for Sports positioning and deciding scale (procedural knowledge) had more chance to reach professional soccer than players who scored low on those scales (Kannekens et al., 2011). In another study, participants watched an occlusion video clip regarding a certain game situation and responded accordingly by circling a decision on a paper. The study consisted of 25 talented and 25 non-talented Australian football players, and the results showed that the talented participants made more accurate decisions (Woods et al., 2016b). These results should be highly relevant for TID in soccer. However, it was found that self or verbal reports measure individual processes, whereas tactical behavior is based upon the interaction between an organism and the environment (Araújo et al., 2010). According to the ecological approach, perception and action continuously interact (Gibson, 1979). Moreover, an athlete during a match, training, or when tested will continuously adapt to the environment and make decisions accordingly. In that regard, the process of identifying talented athletes should take into account an environment in which perception and action are related (Araújo et al., 2009).

In an attempt to create a setting in which perceptual–cognitive skills are measured in a “natural” setting, the Game Insight inDicator (GID) was developed (Savelsbergh et al., 2006, 2010). During the GID, participants react to a positional soccer game shown on a screen in front of the participant. The positional game is occluded before a pass is given toward the participant. The participants move as fast as possible to the position they perceive to intercept the ball after the video was occluded. Savelsbergh et al. (2010) found that skilled amateur youth soccer players were more accurate in moving toward the correct position to intercept the ball than the less skilled players (Savelsbergh et al., 2010). However, the validity of video-based decision making is questioned (Bennett et al., 2019). The latter study underlined the use of sport-specific response actions and a realistic view without removing key contextual information for decision making in a test setting. In addition, the study concluded that within-group differences should be found with a TID tool for practical relevancy.

With current knowledge regarding TID and video assessment tools, the GID should be further examined for practical relevance. Validation of GID could be improved, as no research has examined the GID in relation to *in situ* game insight and game performance. Furthermore, coaches' judgment in regard to perceptual–cognitive skills should be further examined, as it can be highly relevant for TID to know whether coaches can differentiate better and lesser perceptual–cognitive players. In addition, a within-group analysis rather than a between-group analysis should be conducted, as it might reveal more detailed information for successful performance determinants and practical use (Savelsbergh et al., 2005; Bennett et al., 2019). Additionally, the GID was proposed as a TID indicator; nevertheless, no study has been conducted to examine the prospective value of the GID. Therefore, the current experiment expands that of Savelsbergh et al. (2010) by adding four elements: (1) Independent coaches ranked players according to their game insight abilities during a 4-vs.-4 small-sided game (SSG); (2) participants were ranked in both 4-vs.-4 and 11-vs.-11 games using an objective rating system; (3) all participants were elite rather than skilled amateur youth soccer players, all playing at a Dutch elite soccer club; and (4) current soccer level was compared with GID data.

This study frame is different from previous literature, as it examines elite players in their youth and at the expertise level. To our knowledge, this is the first study that examines the prospective value of perceptual–cognitive skills—measured with an interactive video assessment tool—while also taking the judgment of coaches into account. Thereby, the aim of the present study is 2-fold: (1) to establish within-group differences for game insight in elite youth soccer players; thereby, validation of the GID could be improved by examining both subjective as objective perceptual–cognitive skills; (2) prospective value of perceptual–cognitive skills is examined by following the trajectory of the participants.

According to previous literature, expert athletes can extract more relevant information than less-expert athletes (Mann et al., 2007; Savelsbergh et al., 2010). Research also showed that early recognition of visual information could lead to better anticipation on the part of experts during given situations (Abernethy and Russell, 1987; Helsen and Starkes, 1999; Williams and Elliott, 1999; Savelsbergh et al., 2002, 2005; Vaeyens et al., 2007). Therefore, it is hypothesized that the GID can differentiate between talented and less-talented players, even within a highly homogenous elite group and at a later age.

## Materials and Methods

### Participants

Fourteen youth soccer players playing in the U12 or U13 team (mean age 12.2 years, SD = 0.5) for an elite youth academy in the Netherlands participated in the study. The self-reported mean age, height, and body weight were, respectively, 12.2 years (SD = 0.13), 149 cm (SD = 2.0), and 37.8 kg (SD = 1.5). Their self-reported soccer experience was 6.8 years (SD = 1.0) and experience at an elite youth academy (M = 3.6 years, SD = 2.3). Coaches associated with the club but independent from the players were asked to watch and rank the players for game insight. This minimized the prior knowledge the coaches had about the players. All coaches were qualified trainers working with elite level players. Before the research, trainers and club management received an explanation of the measurements, the risks, and the benefits of the study. Parents or guardians were asked to sign an informed consent before the measurements. The study was conducted in agreement with the local university's ethics committee.

### Materials

**Figure 1:**
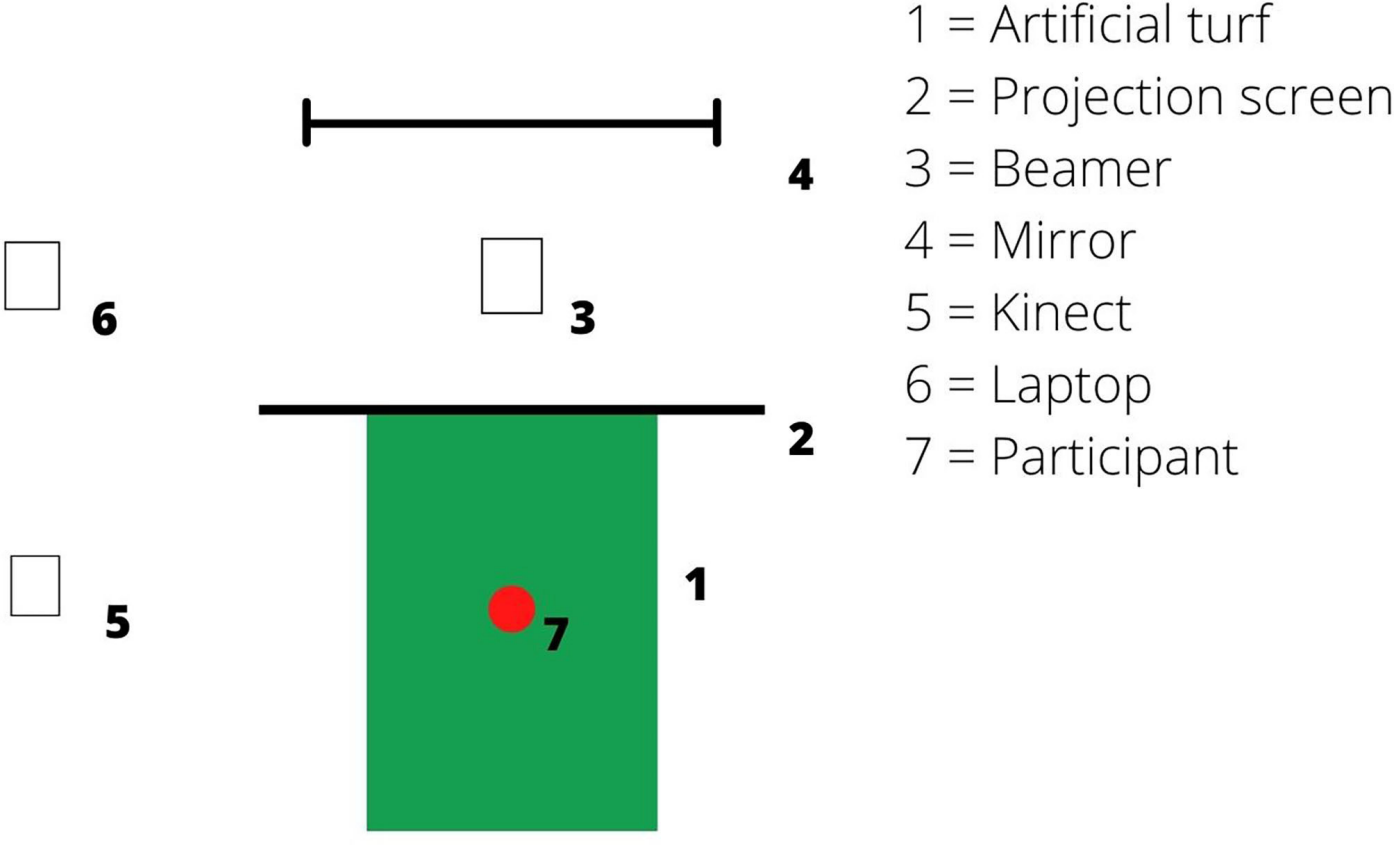
Bird-eye view of the video task setup.

Video cameras recorded the clips for the GID, the regular games, and the SSGs. Video footage was projected on a 2.4 by 2.4 m screen with a video projector (Benq MX 717). An artificial turf mat was placed on the floor in front of the screen, enabling the players to wear their regular soccer shoes. The participant's positions were recorded using a Microsoft Xbox 360 Kinect sensor with coordinates saved on a laptop (HP EliteBook 8570W). The Kinect has been validated for large body movements (Geerse et al., 2015). All measurements were performed in one of the change rooms within the club's facilities (Figure 1).

#### Game Insight inDicator Clips

**Figure A1:**
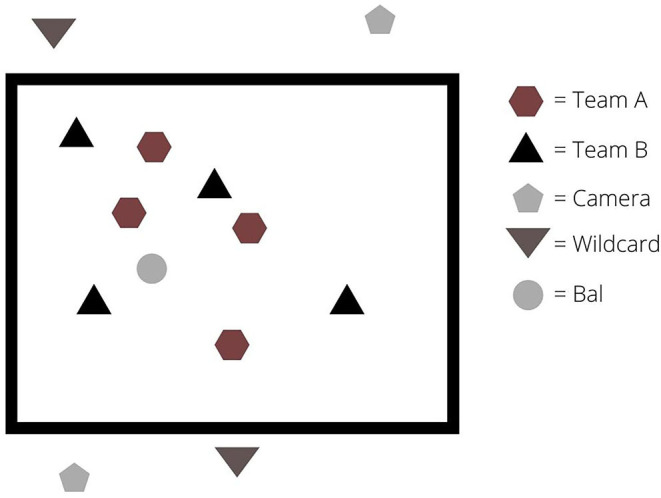
A 4-vs.-4 position game played by peer-aged players with two wildcard players at the side. It was played on an 18.3 by 18.3-m field. Four games of 5 min each have been recorded by two cameras positioned on a wildcard's side line.

GID footages were recordings of a position game played on an 18.3 by 18.3 m field by 4-vs.-4 peer-aged players with additional two wildcard players. Both wildcard players were lined up on opposite sidelines, returning the ball to the team originally playing the ball. This created a 6 vs. 4 advantage for the team in ball possession. Four games of 5 min each were recorded by two cameras positioned on a wildcard's sideline (see Figure A1). Video clips were eligible for the GID if the ball was played in the general direction of one of the cameras, without any obstruction by a player. Based on previous research, three different occlusion types were specified (Abernethy and Russell, 1987; Savelsbergh et al., 2006):

- Clip occludes 80 ms before football contact (−80 ms) (screen turns black)
- Clip occludes at football contact (0 ms) (screen turns black)
- Clip occludes 80 ms after football contact (+80 ms) (screen turns black)

#### Questionnaire

Participants self-reported anthropometrics (height and weight), soccer experience (at the current and previous clubs), time participating in other sports, and time spent playing outdoors. The questionnaire consisted of the following questions:

- Date of birth?
- What are your height and weight?
- Which position do you play? (goalie, defender, midfielder, or forward)
- How many years have you played soccer in general?
- How many years of those have you played at this club?
- How many hours a week do you practice for soccer?
- How many hours a week do you spend playing outdoors?

### Procedure

#### Rating Game Insight Abilities

Participants were involved in five 6-min SSGs with 2-min breaks between the games. SSGs are shown to replicate situations and skill requirements of regular match play (Owen et al., 2004; Rampinini et al., 2007). Thereby, varying the pitch size or amount of players can provide different training responses for physical, physiological, or perceptual loads. For instance, reducing the number of players results in a significant increase of ball contacts and tactical decisions performed by a single player (Owen et al., 2004; Jones and Drust, 2007; Duarte et al., 2009). There is, however, a lack of consistent use of SSG design (Hill-Haas et al., 2011). The current study uses the SSG design 4-vs.-4 with five 6 min SSGs and 2 min breaks in between, as it was part of the training to increase physiological demands. This 3:1 work/rest ratio is within the range of previous research regarding SSG (Hill-Haas et al., 2011). All teams consisted of four players, and the composition of the teams was shuffled every game to prevent social preference bias. The field measurements were 20 by 40 m with a small goal placed on either side. Compared with previous literature, this pitch size was relatively long for an SSG (Hill-Haas et al., 2011). This caused increased physiological demands without affecting the technical demands (Owen et al., 2004). In addition, this longer and narrow pitch was assumed to increase longitudinal inter-team distance and decrease lateral inter-team distance (Frencken et al., 2013). Therefore, it was assumed that there would be more longitudinal space and, thereby, more situations to play the ball forward or backward. These *in situ* situations were examined with the GID videos.

For both the U12 and U13 teams, four independent coaches viewed the video of the five SSGs and separately ranked the players. In total, eight coaches ranked the players according to their game insight abilities. The definition used for game insight is “to act appropriately with the given situation.” Coaches gave participants a score on a scale from one to three, one for performing best and three for performing worst. All coaches judged every player in their allocated age group at least twice, with a maximum of four times. All participants were ranked 10 times. The participants rated a “1” 70% or more were placed in the *highly talented* group. The participants rated a “1” 30% or less were placed in the *less talented* group. This rating was used as the gold standard of game insight in this study. Accordingly, the performance of participants on the GID and for other variables was analyzed when comparing the highly and less talented group.

#### Video Occlusion Task

The GID consisted of 60 occlusion clips, proportionally distributed for three occlusion conditions (Côté et al., 2003). The first three trials were disregarded to allow the participants to familiarize themselves with the setup. The task required participants to anticipate the trajectory of the oncoming ball when visual information was occluded. Before the task, the participants received a brief explanation about the experimental setup and instructions regarding the GID. The task was to be considered a (real-)life-size video game instead of a scientific experiment. In each trial, the ball was played toward the participant or to their left or right. The participants were instructed to receive or intercept the ball, which could be achieved through lateral movements in front of the screen. Before each trial, participants were instructed to return to the center of the screen to prepare for the next trial. When the video clip ended, the participants' positions were recorded with the Kinect for an additional 3 s to capture any movement after occlusion. No feedback was given about performance, but continuous supportive comments were made to aid the participant in maintaining their focus. The occlusion score was determined by calculating the percentage of correct responses to receive or intercept the ball. For each condition (19 trials per condition), the maximum score was 33%. So, for instance, at one condition, 12 correct responses correspond with 21%.

#### Game Performance

Game performance was analyzed during SSGs and regular games. The same footage in which the coaches viewed and ranked the players for game insight was used for analyzing the game performance. Footage of regular competition games was acquired during regular games, which were held in accordance with the Royal Dutch Soccer Association rules on a 60 by 100 m field. The first 30 min of each half was analyzed independently by two notation analysts. Any additional time was excluded from the analysis. Table 1 consists of all variables and the definitions for which the players were rated. The mean percentage of the sum of successful actions was used as an indicator for game performance. Whether the pass, pass reception, or interception was successful depended on the outcome. An action was deemed successful if the team remained in possession or regained possession from the opponent.

**Table 1:** Definition of parameters used for the rating of game performance.

**Parameter**	**Definition**
Receive	Player gains or attempts to gain control of the ball to retain possession
Well-received	The player successfully gains or attempts to gain control of the ball to retain possession
Defensive pressure/interception	Preventing an opponent's pass from reaching its intended destination or put pressure on a player in possession of the opponent
Successful defensive pressure/interception	Successfully preventing an opponent's pass from reaching its intended destination or put successfully pressure on a player in possession of the opponent
Number of passes	Pass: Player in possession sends the ball to a teammate (e.g., using the foot, thigh, or chest; using various techniques such as ground, lofted, chip, flick, or volley; over short or long distances)
Number of passes correct	Amount of successful passes given by a player in a match
Number of passes forward	Amount of passes forward given by a player in a match
Number of passes forward correct	Amount of successful passes forward given by a player in a match

### Dependent Variables, Data Analysis, and Statistics

To conduct analyses for the GID, all x,y,z-coordinates and frame numbers were extracted from the Kinect data. The starting position was determined by determining the participants' mean position over the first 50 frames. Participants' position at the moment of occlusion was used as the final position. A movement of more than 15 cm from the relative starting position was arbitrarily chosen to differentiate among a left, right, or middle response. The dependent variable for GID was the percentage of correct responses. The dependent variable for the SSG and competition game was the mean percentage of the sum of successful first touches, passes, forward passes, and interceptions.

Statistical analyses were performed with SPSS (IBM SPSS Statistics 26.0). An independent *t*-test was used to compare the highly and less talented groups for rating by coaches, rating game performances, and the questionnaire. A mixed analysis of variance design, with between-subject factor groups, was performed to evaluate coach rating for the group [highly talented (HT) vs. less talented (LT)] and occlusion time (−80 vs. 0 vs. +80). If Mauchly's test was not significant (*p* > 0.05), the sphericity assumption was accepted; if not, a Greenhouse–Geisser correction was used. *Post hoc* pairwise comparisons were done with independent *t*-tests to examine if there was a significant interaction effect.

## Results

### Coach Rating

Based on the mean coach ratings, HT and LT participant groups were created. The HT group consisted of five players rated a “1” 70% or more (*N* = 5). The LT group consisted of eight players who were rated a “1” 30% or less. This excluded one player from further analysis. The overview per coach and player is found in Table 2.

**Table 2:** (A) Overview coach ratings U12 players. (B) Overview of coach ratings U13 players.

	**Trainer 1**					**Trainer 2**					**Trainer 3**					**Trainer 4**						**“1”%**
**Players U12**	**L1**	**L2**	**L3**	**L4**	**L5**	**L1**	**L2**	**L3**	**L4**	**L5**	**R1**	**R2**	**R3**	**R4**	**R5**	**R1**	**R2**	**R3**	**R4**	**R5**	**Mean**	
**A**																						
A	2					2						3	3	3	3		3	3	3	3	2,8	0
B	3		3	3	3	3		3	3	3		3					3				3	0
C				3					2		1	2	1		3	2	2	2		1	1.9	30
D		2	1		1		2	1		1	3			1		1			1		1,5	70
E		3		3	3		3		2	3	2		2			3		1			2,5	10
F	1					1						2	1	1	1		1	1	1	1	1,1	90
G	1		1	1	1	1		1	1	1		1					1				1	100
H	2	2				2	2						2	2	1			2	2	2	1,9	10
	**Trainer 5**					**Trainer 6**					**Trainer 7**					**Trainer 8**						**“1”%**
**Players U13**	**L1**	**L2**	**L3**	**L4**	**L5**	**L1**	**L2**	**L3**	**L4**	**L5**	**R1**	**R2**	**R3**	**R4**	**R5**	**R1**	**R2**	**R3**	**R4**	**R5**	**Mean**	
**B**																						
I		1			1		2			2	2		3	3		2		3	3		2,2	20
J		1	1	2			3	1	1		1				1	2				1	1,4	70
K	3	3	2		2	3	3	2		1			2					2			2,3	10
L			1	1	1			1	1	1	1	2				1	2				1,2	80
M	1	2				2	2						1	1	1			1	1	1	1,3	70
N	1			1		2			2			1	1		2		1	1		2	1,4	60

A significant difference (*p* < 0.001) was found for the ratings of the players across the HT group (M = 1.2, SD = 0.16) and LT group (M = 2.25, SD = 0.52) (Table 3). Also, significant differences were detected in the game performance for both the 4v4 and the 11v11 games between the HT group (M = 85.84, SD = 8.2; M = 86.82, SD = 6.8) and the LT group (M = 69.7, SD = 5.8; M = 63.26, SD = 9.3).

**Table 3:** Relevant variables between HT and LT group, *significant (*p* < 0.05).

**Variable**	**HT Group (*N* = 5) Mean (SD)**	**LT Group (*N* = 8) Mean (SD)**	***T*-value (*p*)**
Rating by the coaches	1.2 (0.16)	2.25 (0.52)	4.3 (<0.000)*
Game performance in 4v4	85.84 (8.2)	69.7 (5.8)	−4.2 (<0.001)*
Game performance in 11v11	86.82 (6.8)	63.26 (9.3)	−4.9 (<0.005)*
Hours spent playing outdoors	10.2 (2.8)	7.3 (3.8)	−1.5 (0.152)

**Figure 2:**
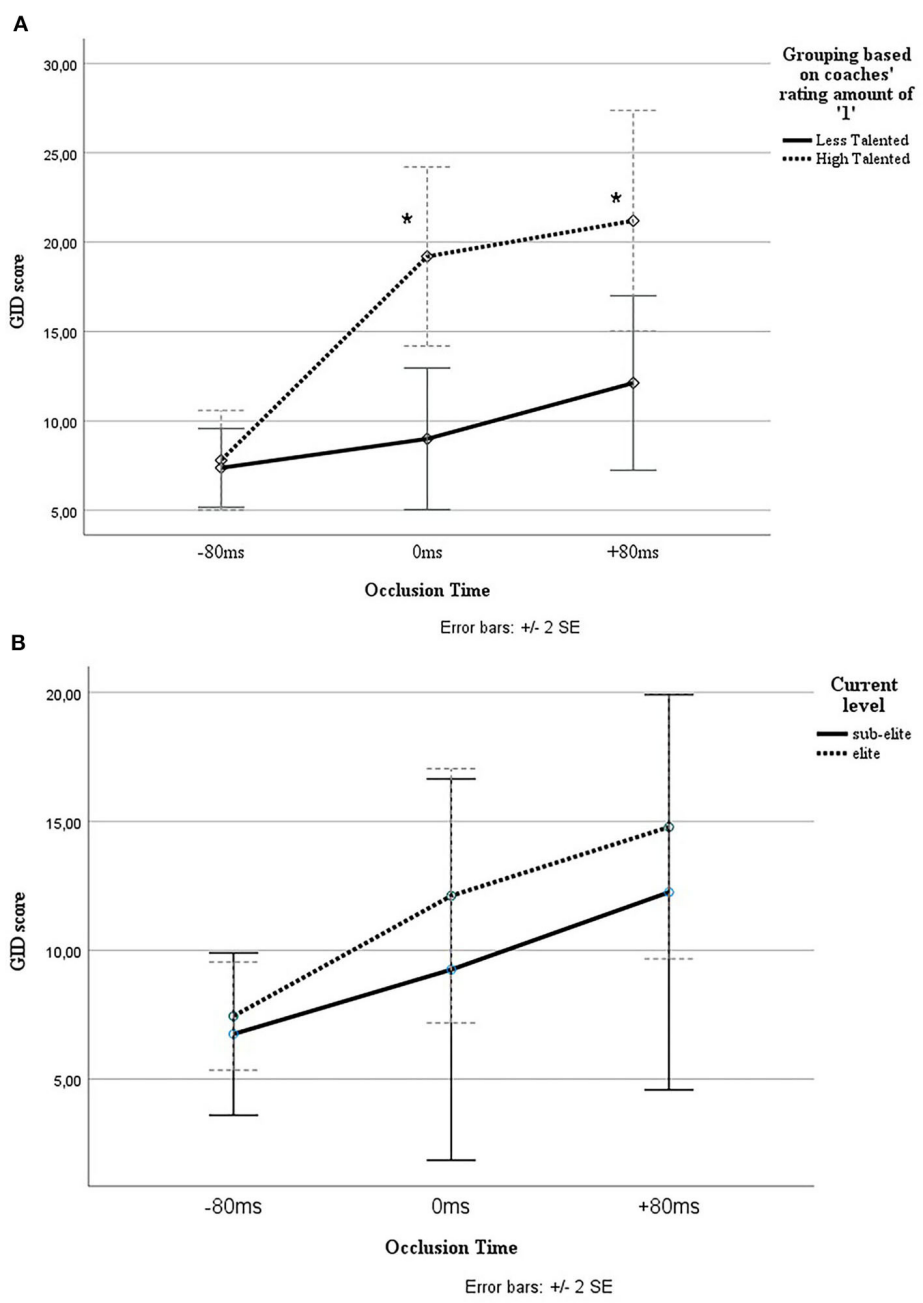
**(A)** Interaction effect for the Group *Occlusion for groups based on coaches rating. *Significant (*p* < 0.05). **(B)** Interaction effect for the Group * Occlusion for groups based on current level.

The group (HT vs. LT) × Occlusion (−80 vs. 0 vs. +80) testing was carried out. The sphericity assumption was accepted due to non-significant Mauchly's test (*p* > 0.05). For the main factor of the group, no significant effect was found *F*_(2, 11)_ = 2.8, *p* = 0.106. The main effect of occlusion was significant *F*_(2, 22)_ = 5, *p* = 0.016, $\eta_{p}^{2}$ = 0.313, whereas the analysis revealed a significant interaction between group and occlusion *F*_(4, 22)_ = 3.4, *p* = 0.025, $\eta_{p}^{2}$ = 0.385 (Figure 2A). An independent *t*-test for GID score showed a significant difference, *t*(11) = −3.2, *p* = 0.009, between score on 0-ms clips for talented (M = 19, SD = 6.4) and less talented group (M = 9, SD = 5). Also, a significant difference, *t*(11) = −2.3, *p* = 0.04, was found between score on +80-ms clips for talented (M = 21, SD = 6) and less talented group (M = 12, SD = 7). For total occlusion score, there was also a significant effect (*p* < 0.05) found for HT group (M = 48.2 SD = 12.6) compared with the LT group (M = 28.4, SD = 13.6).

### Seven Years Later

Seven years after the original measures, 9 of the 14 players still played soccer at a high level (i.e., in the first league for their age). The playing levels of the participants (mean age of 19 years) are reported in Table 4.

**Table 4:** Current soccer level 2019/2020.

**Participant**	**Group**	**Current soccer level**
F	HT	1
L	HT	3
M	HT	1
G	HT	Quit
D	HT	4
J	HT	1
A	LT	3
B	LT	1
C	LT	1
E	LT	1
H	LT	5
I	LT	1
K	LT	1
N	MT	1

*Playing level 1 is the highest possible level—still playing in a premier club at the highest division—whereas level 5 is playing in the fourth division*.

Three out of the six original HT players were playing at the highest level (1), one was performing at the third-best level (3), one at the fourth-best level (4), and one player, unfortunately, had to quit soccer due to reasons other than his soccer skills and was therefore excluded for further analysis. Five of the seven players—originally classified as LT players by the coaches—also played at the highest level. Additional analysis found no significant effect on the main factor group (*p* = 0.568) between sub-elite and elite players (Figure 2B). Also, no significant effect was found for interaction effect between group and occlusion (*p* = 0.634) between the elite (*N* = 9) compared with sub-elite (*N* = 4).

## Discussion

The purpose of this study was to verify and expand findings by Savelsbergh et al. and improve the validation of the GID. In previous research, the GID was validated as a differentiator between skilled amateur youth players (Savelsbergh et al., 2010). However, the capabilities of video-based decision-making have been questioned (Bennett et al., 2019). The current findings confirm our hypothesis that talented and less-talented youth participants can be identified on the basis of their performance on the occlusion task. Within a highly homogenous elite group, the HT group outperforms the LT group for occlusion task and game performance (Table 3, Figure 2A). This also indicates that independent elite coaches can differentiate HT players and LT players within only 30 min of SSG play. Further, these coach ratings are supported by the significant difference between the two groups revealed by objective notational analyses of the 4-vs.-4 and 11-vs.-11 game performance.

A significant interaction effect for occlusion and group was found. A clear difference was observed visually between the HT and LT groups (Figure 2A). This is in line with earlier literature stating that expert athletes can extract more relevant information than less-expert athletes (Mann et al., 2007; Savelsbergh et al., 2010; Roca et al., 2012). In the current study, the −80 and +80 ms occlusion scenarios do not (quite) differentiate between the HT and LT groups. From the literature, it is well-known that adult experts are capable of extracting relevant cues at 160 ms before final ball contact and that −80 ms occlusion trials show to be a good occlusion scenario to discriminate within expert adults (Abernethy, 1990; Mann et al., 2007). The current study showed that the −80 ms occlusion trials were too difficult for the young participants. Explanations that may account for this: (1) the cognitive functioning of the young brain has not been fully developed and lacks the processing speed; (2) the participants do not have the amount of experience of adults and lack a reference framework to compare the current situation with; (3) It may yet not be necessary for youth players to make anticipation for that time constraint (Weissensteiner et al., 2008). In addition, it has been suggested that children need more time to decide during an occlusion task (Savelsbergh et al., 2010). The +80 and 0 ms occlusion scenarios seem to be good methods to discriminate within a highly talented youth soccer group between HT and LT players. The uptrend in Figure 2A for LT players at +80 ms trials reflects the elite level of the participants. Future research should indicate whether +80 ms scenarios discriminate more between sub-elite and elite.

By using both subjective and objective measurements, the current study presents a broad view of game insight. Elite coaches created both the HT and LT groups on their game insight abilities. Between both groups, a significant difference in game performance, measured by objective analysis, was observed. Current findings, therefore, note the importance of game insight during a game performance, whereas game performance depends on multidisciplinary skills (Larkin and O'Connor, 2017). GID might, therefore, be an important tool to indicate game insight and game performance. Nevertheless, there might be a discrepancy between the GID and real gameplay. Game insight during gameplay is suggested to be characterized by explorative behavior, which increases the rate of successful actions of soccer players (Jordet et al., 2013; McGuckian et al., 2018). A recent study, thereby, proposes that players who have knowledge regarding their surroundings can make better actions accordingly (McGuckian et al., 2018). GID measures perceptual–cognitive skills in the frontal plane and, therefore, might require less explorative behavior than the *in situ* gameplay, which requires a three-dimensional view (Vaeyens et al., 2008). The latter suggests that the GID is less representative of real gameplay and, therefore, might not be suitable for TID regarding perceptual–cognitive skills (Shim et al., 2005; Bennett et al., 2019). However, identifying talent should be executed in an environment with an intact coupling between perception and action (Araújo et al., 2009). In addition, the task should be representative of soccer matches and require the execution of sport-specific skills (Travassos et al., 2013; Bennett et al., 2019). During the GID, participants are tasked to receive the ball and not give a pass afterward or make a certain action. Therefore, the GID creates a decision-making situation that is encountered by players during regular games. In addition, the GID provides environmental information such as teammates and task requirements, which decision-making tasks should consist of Araújo et al. (2006) and Mann et al. (2007). The GID also contains decision and processing speeds required during a competition, which should be reflected during practice sessions (Farrow and Raab, 2008). Overall, the GID gives a representative view of decision making and might be a more suitable tool to measure tactical decision making than verbal or written tests (Van Der Kamp et al., 2008; Araújo et al., 2009, 2010; Travassos et al., 2013). The latter is supported by the findings that *in situ* performance of SSGs did not relate to self-reported procedural data (Nortje et al., 2014). This is in line with the idea of Van Der Kamp et al. (2008) that an experimental study regarding decision making should require participants to act rather than to write or communicate. The GID, however, is performed in a controlled laboratory setting. A limitation, therefore, is the presence of a discrepancy of perception–action coupling between *in situ* and the controlled setting. The perception in a laboratory setting is found to be different, whereas the player in the natural setting has to perform the required action according to the perceived information (Dicks et al., 2010). The intention with the GID is only to move toward a position to intercept the ball without performing the interception. Current results, however, indicate that occlusion scores differentiate for *in situ* game insight. Nevertheless, further research should be undertaken to compare occlusion scores with explorative behavior during real gameplay to increase the validity of the GID and *in situ* decision making.

### Seven Years Later

Longitudinal data show that five of the seven players—which were originally classified as LT players by the coaches—played at the highest level at mean age 19 years. This indicates how difficult it is to predict talent development. Current data show that trainers should not base their decision on (de)selecting exclusively on GID score. Nevertheless, although there was no significant difference for longitudinal data, GID is still very promising as the elite group scored better than the less group (Figure 2B). The improvement of the less group might be caused by the nature of the soccer club, i.e., having a long-distance plan with their youth players. During the years of training, the “less talented” players could have increased their perceptual–cognitive, and other skills, with the number of training hours at the elite level. GID could, therefore, be a valuable tool for measuring and evaluating the development of perceptual–cognitive skills. Future research should examine longitudinal data for the GID with a larger sample size to indicate the usefulness of the GID for talent prediction.

### Additional Information

This section contains findings that are outside the scope of this study. Nevertheless, it contains important and useful information for TID and development. A growing body of evidence arises that children and young adolescence are spending more time indoors and becoming more sedentary (Hallal et al., 2012; Tremblay et al., 2014, 2016). A recent study by Anselma et al. (2020) found that the decrease of physical activity results in a decrease of movement speed, flexibility, and trunk, leg power for children of 10–12 years compared with 10 years before. Another recent study with 2,543 children showed that the physical activity of children with mean a mean age of 10 years was 9.06 hours per week (SD = 5.10) (Rodriguez-Ayllon et al., 2020). In comparison, the physical activity per week of the soccer players in the current study was considerably higher. With organized sports hours per week and hours playing outside taken into account, the self-reported total physical activity of the talented players was 14.7 h and, for the lesser counterparts, 11.8 h per week. The discrepancy with the non-expert peers might result in an ongoing advancement for elite players, whereas a decrease in physical activity decreases characteristics needed for soccer (Anselma et al., 2020). In addition, outdoor play improves responses to new and challenging environments and social skills (Pellegrini and Smith, 1998; Pellegrini et al., 2007). Furthermore, the extra hours of practice could play a significant role in the development of the players (Côté et al., 2003; Memmert et al., 2010). The motor development of future athletes could, therefore, be hindered and as a consequence the gap between expert elites and novices increases. Future research should take the latter into account and examine whether the gap between novices and expert athletes increases as this might be concerning for the overall level of future athletes.

Overall, the current findings indicate that the GID is a useful indicator for anticipating the ball's trajectory and destination at age 11–13. Although no clear relationship with future performance was found, the GID can be a valuable tool to measure and evaluate perceptual–cognitive skills to examine the process of talent development. Yet, *in-situ* game insight measurement and coaches evaluation had some limitations. Future research should, therefore, evaluate game performance by taking explorative behavior into account. Furthermore, the current findings point to the need to consider the subjective opinions of expert coaches for talent identification. This is in line with recent literature that the judgment of coaches regarding soccer skill rating discriminates between different skill groups (Hendry et al., 2018). Although there is a growing body of literature regarding objective talent predictors, coach judgments are still important and should not be neglected in the selection and deselection of soccer players (Höner and Votteler, 2016). Nevertheless, coaches' evaluations of game insight might be biased. Therefore, it is suggested that future research should implement the actuarial judgment of coaches to improve the evaluation of game insight performance *in situ*. The actuarial judgment gives more rules for the judgment and decreases the chance of biased judgments (Christensen, 2009; Den Hartigh et al., 2018). Combining both actuarial judgments and the GID could provide a broader understanding of the game insight of soccer players.

## Data Availability Statement

The raw data supporting the conclusions of this article will be made available by the authors, without undue reservation.

## Ethics Statement

Ethical review and approval was not required for the study on human participants in accordance with the local legislation and institutional requirements. Written informed consent to participate in this study was provided by the participants' legal guardian/next of kin. Written informed consent was obtained from the minor(s)' legal guardian/next of kin for the publication of any potentially identifiable images or data included in this article.

## Author Contributions

GS conceptualized the study. DT and TdJ contributed to the software and data collection. TdJ contributed to the methodology and writing of the original draft. GS reviewed and edited the manuscript and supervised the study. All authors contributed to the article and approved the submitted version.

## Conflict of Interest

The authors declare that the research was conducted in the absence of any commercial or financial relationships that could be construed as a potential conflict of interest.
